# Lemierre syndrome: a hidden complication of sore throats

**DOI:** 10.1186/s12245-023-00524-x

**Published:** 2023-07-24

**Authors:** Naheel A. AlAmer, Wasan F. AlMarzouq

**Affiliations:** 1 Department of Family and Community Medicine, College of Medicine, Imam Abdulrahman Bin Faisal University, Dammam, Saudi Arabia; 2 Department of Otolaryngology-Head and Neck Surgery, College of Medicine, Imam Abdulrahman Bin Faisal University, Dammam, Saudi Arabia

**Keywords:** Lemierre syndrome, Thrombophlebitis, *Klebsiella pneumoniae*, Neck swelling, Septic emboli, Antibiotic stewardship

## Abstract

**Background:**

Lemierre syndrome is a rare but potentially life-threatening condition characterized by septic thrombophlebitis of the internal jugular vein, most commonly caused by *Fusobacterium necrophorum*.

**Case presentation:**

A 46-year-old woman with no significant medical history presented with generalized neck swelling and fever. Despite seeking medical attention at multiple outpatient clinics for upper respiratory symptoms lasting 10 days, she only received symptomatic treatment for acute viral pharyngitis. A computed tomography (CT) scan of her neck revealed thrombophlebitis in the left internal jugular vein, and subsequent cultures indicated the presence of *Klebsiella pneumoniae*. The patient’s oxygen saturation levels suddenly dropped, and the CT scan identified bilateral peripheral consolidation areas in both lungs, consistent with septic emboli. These findings were consistent with a diagnosis of Lemierre syndrome. The patient was treated with 2 weeks of intravenous piperacillin/tazobactam and vancomycin, as well as anticoagulation therapy using heparin, and her symptoms resolved completely.

**Conclusion:**

This report presents an unusual occurrence of Lemierre syndrome caused by *K. pneumoniae*, a less frequently encountered causative pathogen in patients without diabetes mellitus. The case highlights the significance of timely and appropriate antibiotic use to prevent potential complications.

## Background

Acute upper respiratory tract infections are a common reason for primary care visits [1]. However, despite the fact that most of these infections are caused by viruses, overuse of antibiotics has become a significant concern in recent years due to the risk of antibiotic resistance [2]. It is crucial, therefore, to use antibiotics judiciously [3]. However, withholding antibiotics when clinically indicated can lead to life-threatening complications [4]. In this report, we present a case of Lemierre syndrome, which is characterized by internal jugular vein thrombophlebitis with septic emboli, in a middle-aged woman with an upper respiratory tract infection who was not initially treated with antibiotics.

Lemierre syndrome, also known as the “forgotten disease,” was first described in the 1930s and is now a relatively rare condition [5, 6]. Despite this, it remains an essential diagnosis to consider, as it can be life-threatening if not promptly treated [5]. *Fusobacterium necrophorum* is typically the causative agent [6, 7], but *Klebsiella pneumoniae* was responsible for Lemierre syndrome in this case.

## Case presentation

A 46-year-old female patient presented to the emergency department with complaints of fever and swelling in her neck. The swelling had been present for 2 days and was accompanied by skin redness and limited mobility in her neck. It was not associated with stridor, dysphagia, or odynophagia. There was no history of trauma, skin rash, weight loss, or night sweats. The patient had no significant medical history and reported no history of smoking, alcohol consumption, or recreational drug use. The family history was also unremarkable.

Prior to this emergency department visit, the patient had sought medical attention from several family medicine physicians for symptoms of an upper respiratory tract infection, including severe sore throat and cough, which had begun 10 days earlier. As the presumptive diagnosis was acute viral pharyngitis, the patient had only received symptomatic treatment and had not been prescribed antibiotics. Despite the lack of improvement in symptoms, no laboratory investigations were performed during these visits.

During the physical examination, the patient appeared ill and was shivering. Vital signs revealed a fever of 38.5 °C, tachycardia of 110 beats/min, and tachypnea of 22 breaths/min. Blood pressure was normal at 115/80 mmHg, and oxygen saturation was 99% on room air. Intraoral examination revealed an erythematous pharyngeal wall with no evidence of tonsillar exudates. The left lateral aspect of the neck showed extensive erythema and swelling, and multiple enlarged lymph nodes were identified. Examination of the cardiopulmonary systems did not reveal any abnormalities.

Initial laboratory investigations were performed, revealing leukocytosis (17,500 cells/μL) with 80.5% neutrophils, a hemoglobin level of 13.2 g/dL, and a platelet count of 460,000 cells/μL. The C-reactive protein (18.5 mg/dL) and erythrocyte sedimentation rate (85 mm/hour) were markedly elevated. The renal, liver, and electrolyte panels were within normal limits. Random blood sugar (105 mg/dL) and hemoglobin A1c (5.6%) levels were normal.

**Fig. 1:**
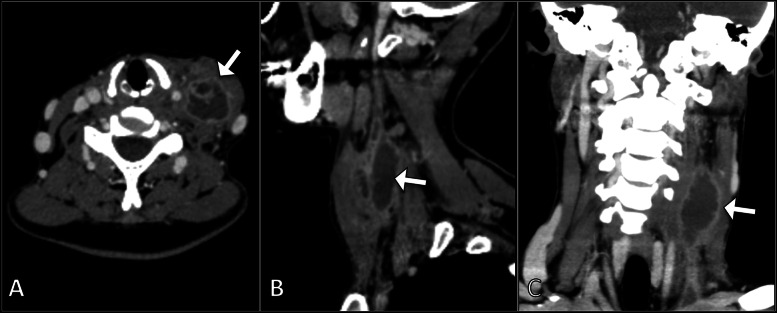
CT images of the neck in axial (**A**), sagittal (**B**), and coronal (**C**) planes demonstrate a distended caliber of the left internal jugular vein with an enhancing wall and intraluminal filling defect consistent with thrombophlebitis (arrows)

A contrast-enhanced computed tomography (CT) scan of the neck demonstrated a distended caliber of the left internal jugular vein with an enhancing wall and intraluminal filling defect consistent with thrombophlebitis (Fig. 1). This was associated with fat stranding within the left carotid sheath and the left lateral aspect of the neck, as well as inflammatory soft tissue tumefaction. Multiple enlarged lymph nodes were observed bilaterally in the submental, submandibular, and internal jugular chain groups. Otherwise, both carotid arteries and the right jugular vein were normal in course and caliber.

The patient was placed on intravenous antibiotic therapy with piperacillin/tazobactam and vancomycin, and anticoagulation therapy with heparin was started. Subsequently, the patient showed remarkable clinical improvement, with resolution of the fever. Both blood and throat cultures drawn at admission revealed positive growth of *K. pneumoniae*. Furthermore, the thrombophilia workup, including the activated protein C resistance test, antithrombin assay, protein C and protein S assays, lupus anticoagulant assay, prothrombin gene mutation test, and homocysteine level, revealed no abnormalities.

**Fig. 2:**
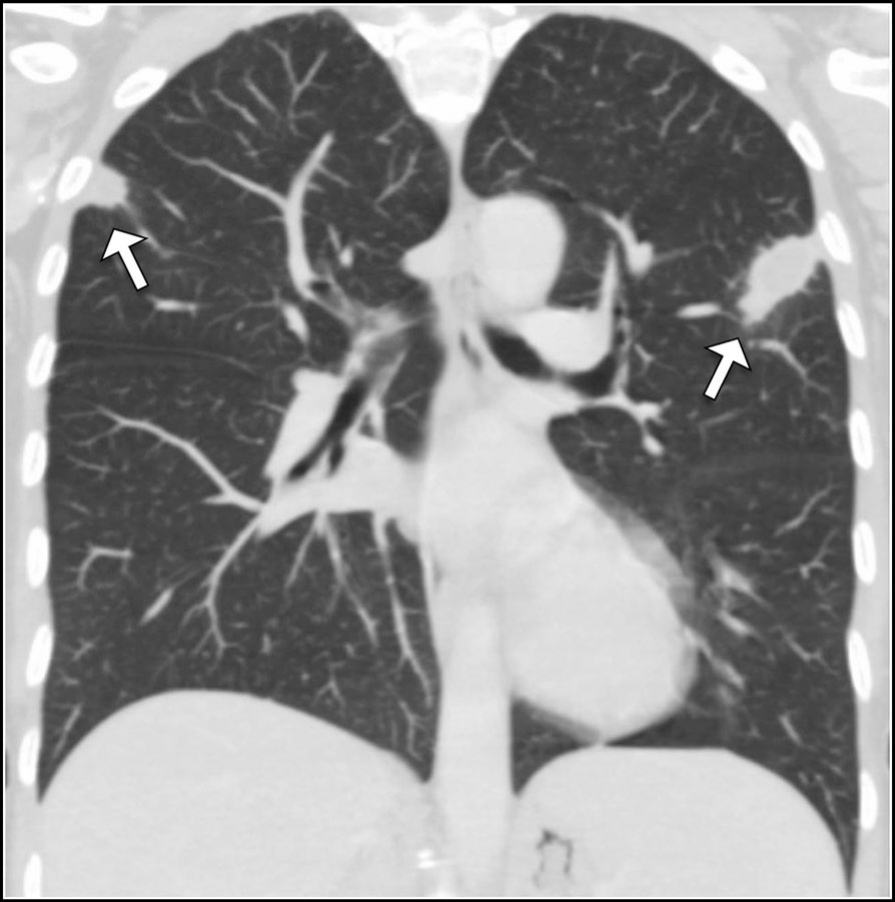
Coronal CT image of the chest shows peripherally located wedge-shaped consolidations (arrows) in the upper lobe of both lungs. These findings support the diagnosis of septic emboli in the presented case of Lemierre syndrome

On the 5th day of hospitalization, the patient experienced a sudden decrease in oxygen saturation to 90% on room air. A CT pulmonary angiography scan was performed, which revealed wedge-shaped areas of consolidation in the upper lobes of both lungs located peripherally (Fig. 2). Given the context of internal jugular vein thrombophlebitis, these findings were consistent with septic emboli. No filling defects were observed within the pulmonary vasculature to suggest acute pulmonary embolism. Furthermore, echocardiography revealed no evidence of thrombus or vegetation.

After 2 weeks of antibiotic therapy, the patient’s symptoms completely resolved. The white blood cell count and other inflammatory markers returned to normal levels. Repeated blood culture showed no significant bacterial growth. The patient was discharged with no active issues.

## Discussion

The case report presents an uncommon complication of oropharyngeal infection in a middle-aged woman with no prior health issues. Lemierre syndrome is a rare but severe condition, characterized by septic thrombophlebitis of the internal jugular vein, commonly resulting from a bacterial infection in the head and neck area. The incidence of Lemierre syndrome is low, with approximately 3.6 cases per million individuals [8]. However, there has been a noticeable rise in the number of reported cases since the 1990s [8], possibly due to increased awareness of the condition and appropriate use of antibiotics [5].

The inappropriate use of antibiotics is a major global public health concern due to the increasing prevalence of antibiotic resistance, which has negative clinical and economic impacts [4]. A study involving more than 1000 adults with respiratory tract infections found that most patients were prescribed antibiotics, with 64% of these prescriptions considered inappropriate [3]. To address this issue, antibiotic stewardship programs have been developed to promote appropriate antibiotic use. However, it is important to note that withholding antibiotics when clinically indicated can also have adverse consequences, such as bacterial sepsis that could be prevented by timely antibiotic treatment [4].

To achieve a balance, clinicians should use clinical decision rules, such as the Centor score for *Streptococcus* pharyngitis, to determine the likelihood of bacterial infection in patients with upper respiratory tract infections [9–11]. However, these rules have limitations and may not always reflect the severity of the patient’s illness or take into account individual factors such as age and comorbidities [9]. Although the patient did not meet the criteria for antibiotic prescription according to the Centor score during initial visits to family medicine clinics, further clinical evaluation and antibiotic therapy were warranted considering the prolonged duration of severe symptoms and timely administering antibiotic therapy could have prevented the development of Lemierre syndrome. Although the patient did not meet the criteria for antibiotic prescription according to the Centor score during initial visits to family medicine clinics, further clinical evaluation and antibiotic therapy should be warranted, considering the prolonged duration and severity of symptoms. Timely administration of antibiotic therapy could have prevented the development of Lemierre syndrome. Although the patient did not meet the criteria for antibiotic prescription according to the Centor score during initial visits to family medicine clinics, further clinical evaluation and consideration of the patient’s prolonged and severe symptoms indicate that antibiotic therapy should be warranted. Timely administration of antibiotics could have prevented the development of severe complications such as Lemierre syndrome.

Lemierre syndrome is typically caused by *Fusobacteria* species, particularly *F. necrophorum*, with other less common pathogens reported including *Bacteroides*, *Enterococcus*, *Proteus mirabilis*, *Streptococcus pyogenes*, and *Peptostreptococcus* [12]. In this case, Lemierre syndrome was caused by *K. pneumoniae*, which is an infrequent pathogen associated with the syndrome, accounting for only 2.5% of cases reported in the literature [13]. However, most *K. pneumoniae*-associated Lemierre syndrome cases occur in patients with poorly controlled diabetes mellitus, which is unlike the present case [7].

Prompt recognition and management of Lemierre syndrome are crucial for reducing mortality rates. In the pre-antibiotic era, Lemierre syndrome had a mortality rate as high as 90% [6]. Modern studies indicate that despite appropriate treatment, the mortality rate can still reach up to 20% [5]. The primary treatment for Lemierre syndrome is prolonged antibiotic therapy, and if medical therapy fails, surgical drainage of abscesses may be necessary [5]. The use of anticoagulation therapy remains a topic of debate and lacks a clear consensus [14]. While some studies suggest that anticoagulation may prevent thrombus extension [15], others show no significant benefit of this approach [16]. Therefore, decisions regarding anticoagulation should be made on a case-by-case basis, taking into account individual patient factors, such as the extent and severity of thrombosis, bleeding risk, and any comorbidities.

## Conclusion

Lemierre syndrome is a rare yet severe complication that can result from oropharyngeal infections. Clinicians should maintain a high level of suspicion for this condition in patients who present with severe neck pain after acute pharyngitis. This case report highlights the critical importance of appropriate antibiotic therapy for severe upper respiratory tract infections that are prolonged or unresponsive to symptomatic treatment. Delaying or withholding antibiotics in the presence of a bacterial infection can lead to life-threatening complications. In our case, Lemierre syndrome was caused by *K. pneumoniae*, which is an infrequent pathogen for this condition, especially in patients without diabetes mellitus. Early recognition and treatment of Lemierre syndrome are crucial for a favorable outcome.

## Data Availability

Not applicable.
